# Recent Studies on Anti-Depressant Bioactive Substances in Selected Species from the Genera *Hemerocallis* and *Gladiolus*: A Systematic Review

**DOI:** 10.3390/ph12040172

**Published:** 2019-11-25

**Authors:** Renata Matraszek-Gawron, Mirosława Chwil, Paulina Terlecka, Michał M. Skoczylas

**Affiliations:** 1 Department of Botany and Plant Physiology, University of Life Sciences in Lublin, 15 Akademicka Street, 20-950 Lublin, Poland; renata.matraszek@up.lublin.pl; 2 Chair and Department of Pneumology, Oncology and Allergology, Medical University of Lublin, 8 Jaczewskiego Street, 20-090 Lublin, Poland; 41497@student.umlub.pl; 3 Department of Diagnostic Imaging and Interventional Radiology, Pomeranian Medical University in Szczecin, 1 Unii Lubelskiej Street, 71-252 Szczecin, Poland; emes@e-post.pl

**Keywords:** daylily, depression, diagnostic imaging, Iridaceae, nervous system agents, neurotransmitters, phytotherapy, Xanthorrhoeaceae

## Abstract

Herbal therapy is a potential alternative applied to pharmacological alleviation of depression symptoms and treatment of this disorder, which is predicted by the World Health Organization (WHO) to be the most serious health problem worldwide over the next several years. It has been well documented that many herbs with psychotropic effects have far fewer side effects than a variety of pharmaceutical agents used by psychiatrists for the treatment of depression. This systematic review presents literature data on the antidepressant activity of representatives of the genera *Hemerocallis* (*H. fulva* and *H. citrina* Baroni, family Xanthorrhoeaceae) and *Gladiolus* (*G. dalenii*, family Iridaceae) and on biologically active compounds and their mechanisms of action to consider the application of herbal preparations supporting the treatment of depression.

## 1. Introduction 

The term “depression” in colloquial language is overused and often equated with sadness, fatigue, or malaise. From the medical point of view, depression is a long-lasting and recurring syndrome affecting the mood and emotions. It is one of the most common pathological conditions persisting over a long time, tending to recur, reducing work capacity, and causing problems in social relations [1,2,3,4].

### 1.1. Depression

#### 1.1.1. Epidemiology with Its Relation to Pathogenesis

Depression is a common mental illness and a major cause of disability worldwide, generating huge economic losses in modern society [5,6,7,8]. It is more widespread in developed than in low- and middle-income countries [7,9]. As reported by the World Health Organization (WHO) [10], approximately 300 million people in the world suffer from depression. The incidence and mortality associated with this disease are high and continue to grow [7,11].

Depression is now ranked the fourth major cause of disability worldwide, after respiratory infections, prenatal conditions, and HIV/AIDS [7]. It has been estimated that depressive disorders will become the second largest global burden by 2030 [12]. Depression is regarded as one of the most common causes of diminution of life span resulting from health disability [13,14,15,16,17,18]. It is a serious disorder affecting up to 21% of the population in some developed countries [7]. Women suffer from depression twice as often as men [7,19,20,21,22,23]. The risk of developing the disease is approximately 10%–25% in women and 5%–12% in men [19,20,24].

Among the latest studies on the causes of depression, those related to diet should be mentioned in this review. A high-fat diet promotes depression-like behavior in mice by suppressing the hypothalamic 3′, 5′-cyclic AMP (cAMP)/protein kinase A (PKA) signaling pathway [25]. Obesity promotes depression, while weight loss reduces the risk of developing this condition [26]. In addition to the quality and quantity of nutrients, the effect of intestinal microflora on the organism appears to be important [27]. In this aspect, there is a potential impact of neurotransmitters, for instance, gamma-aminobutyric acid (GABA), which is known to play a role in mental disorders [28,29]. As suggested in many reports, this fact and the results of studies in mice, e.g., the role of agonists of cannabinoid 1 receptor signaling in the modulation of microglial activity acting through GABAergic interneurons, the issue of the neuroprotective effects of biologically active substances should be taken into consideration [30,31]. Reduced GABA levels have been detected in patients with depression, including women in the postmenopausal period [32]. An important role in providing protection for nerve cells is also played by chaperones described by Kurek et al. [33], who investigated the regulators of glucocorticoid receptor function in an animal model of depression and obesity.

There is no doubt that depression accompanies many human diseases or is in a range of adverse drug reactions. On the other hand, the emergence of this illness has an impact on the results of the treatment of some diseases, for instance, causing inefficiency of the therapy of autoimmune hepatitis and increasing the risk of its relapse and progression [34]. Appropriate treatment of depression gives a bigger chance of curing other diseases.

#### 1.1.2. Symptoms and Their Organic Explanation

The first symptoms of depression (age of onset, AOO) appear most frequently during early adulthood between 20 and 35–40 years of age [35,36,37,38,39,40,41,42]. However, depression afflicts the elderly as well: the first symptoms in more than half of them appear after 60 years of age. A later onset of the disease is associated with a higher risk of suicide than in the group of younger subjects [43,44,45].

Depression is diagnosed at a simultaneous presence of at least two primary and two additional symptoms of the disease persisting for over two weeks [46,47,48,49]. Its primary symptoms include depressed moods occurring every day and persisting for most of the day, loss of interest and/or lack of ability to feel pleasure (anhedonia), loss of energy, and increased susceptibility to fatigue. Additional symptoms comprise loss of self-confidence and self-esteem, feelings of guilt, and recurrent thoughts of death and suicide. There are also problems with memory and concentration, sleep disorders (both insomnia and excessive drowsiness), and changes in activity and appetite [46,47,48,49,50].

Depression affects mood, behavior, and mental health. The organic explanation for these phenomena was found to be based, e.g., on inflammatory processes and mental stress with such mediators as interleukins (mainly IL-6), tumor necrosis factor alpha (TNFα), nuclear factor-*κ*B (NF-*κ*B), and adrenocorticotrophic hormone (ACTH) [51,52]. Moreover, the “macrophage theory of depression” is still being studied [53]. Patients with depression also show symptoms reflecting changes in the neurotransmitters in the central nervous system (CNS), especially noradrenaline (NA) (i.e., norepinephrine, NE), serotonin (5-hydroxytryptamine 5-HT) 5-HT, and dopamine (DA) [54,55,56,57]. Adequate nutrition plays a key role in many aspects of brain functioning [58]. Inadequate quality of the diet can be a modifiable risk factor for depression; for instance, a low concentration of omega-3 acids increases the risk of development of depression symptoms [59]. In the study performed by Bondar and Wiser [60], patients with depression have been diagnosed with a deficiency of folic acid, vitamin B12, zinc, iron, and selenium. An abnormal blood sugar level is associated with an increased incidence of postnatal depression [61].

There are many therapies available for patients with depression and anxiety disorders, e.g., psychotherapy, electroconvulsive therapy, and antidepressant drugs. For safety reasons and given the side effects and the limited efficacy associated with many antidepressants and anxiolytics as well as the low tolerance to these agents, a search for new drugs with lower toxicity and higher treatment efficacy is advisable [42,62,63,64,65,66,67,68,69,70,71].

#### 1.1.3. Neurotransmitters as the Key Pathogenetic Factors

With regard to the above-described interactions on organ systems at the tissue level, particular attention should be paid to the molecular level of intercellular communication in the central nervous system. While glial cells affect neuronal condition and function, especially in the described inflammatory conditions, the neuron–neuron relationships depend mainly on neurotransmitters.

Signal transmission in the nervous system is carried out by neurotransmitters released from neuronal endings in response to a depolarization wave [72]. Over 50 various neurotransmitters exerting several effects on the human organism and mobilizing various types of cells have been discovered so far [73,74]. There are excitatory neurotransmitters such as noradrenaline and adrenaline and inhibitory neurotransmitters, for instance, gamma-aminobutyric acid (GABA) and glycine [75]. Some neurotransmitters released into the synaptic cleft bind are decomposed but partially recovered in the reuptake mechanism [76]. Disorders in the release of neuromediators can lead, e.g., to neurological or mental diseases, such as schizophrenia, Alzheimer’s disease, and Parkinson’s disease. Depressed subjects have been diagnosed with reduced content of neurotransmitters 5-HT, NA, GABA, DA, acetylcholine (ACh) in the CNS [54,56,57,58,59,60,61,62,63,64,65,66,67,68,69,70,71,72,73,74,75,76,77,78,79,80].

The neurobiological causes of depression have not yet been clearly identified. A monoamine hypothesis has been proposed to elucidate the pathogenesis of this disease. It assumes that reduction of the level of monoamine transmitters in the CNS, e.g., 5-HT, NA, and DA, is responsible for the development of depression [54,77,78,79,80]. Dopamine deficiency in the CNS may be a cause of depression and “foggy brain” [81]. Reduced activity of noradrenergic neurons contributes to the development of depression [82,83,84], whereas their increased activity has been observed in manic syndromes [85].

Serotonin represents a group of biogenic amines and is a derivative of tryptophan. There are a number of serotoninergic receptors, e.g., 5-HT1A, 5-HT1B, 5-HT2Ab, 5-HT3, and 5-HT4. The 5-HT1A receptor is involved in the recognition, memory, and learning processes. Activation of this receptor is one of the mechanisms of action of antidepressant drugs [86,87,88,89,90,91]. Wang et al. [56] analyzed publications from 1999 to 2015, presenting the relationship between alterations in 5-HT1A receptors and depression. The authors confirmed the relationship of the pathophysiology of depression with reduced 5-HT1A receptor binding and neurotransmission disorders in the CNS.

The monoamine hypothesis has been supported by the results of studies on the effectiveness of antidepressants. The treatment of depression is primarily based on second-generation antidepressant drugs: selective serotonin reuptake inhibitors (SSRIs) and serotonin-norepinephrine reuptake inhibitors (SNRIs) [90,92,93,94,95,96,97,98,99,100,101,102,103,104,105]. The mechanism of action of this group of drugs consists in strengthening the neurotransmission in the CNS by blocking the reuptake or degradation of relevant monoamine neurotransmitters and increasing their content in the synaptic cleft [75,90,99,106,107,108,109,110].

The validity of the monoamine hypothesis is also confirmed by the efficacy and mechanism of action of first-generation drugs, e.g., monoamine oxidase inhibitors (MAOIs) and tricyclic antidepressants (TCAs). Monoamine oxidase inhibitors block 5-HT, NA, and DA reuptake, thereby elevating their levels in the CNS. TCAs contribute to increased neurotransmission of NA and 5-HT via the same mechanism [80,90,111,112,113,114,115,116].

Reduced GABA synthesis observed in depressed patients is associated with increased anxiety and anhedonia [22,117,118,119,120,121,122]. Normalization of GABA levels in patients treated with SSRIs has been shown to alleviate depression symptoms [22,123,124,125]. Dysfunction of the glutamatergic system is known to contribute to the pathogenesis of depression [120,126,127,128,129,130,131,132]. Ketamine, which blocks the NMDA glutamate receptor, has been demonstrated to have antidepressant properties [133,134,135,136,137,138,139,140]. Elevated concentrations of glutamate in blood serum and cerebrospinal fluid have been detected in depression patients [141,142].

Besides the main (primary) monoaminoergic hypothesis of depression, there are many other approaches to the pathophysiology of this disease, including dysregulation of the hypothalamic-pituitary-adrenal axis and impairment of dopaminergic, cholinergic, glutamatergic, or GABA-ergic neurotransmission. Nevertheless, there is no doubt that the serotonergic, noradrenergic, and dopaminergic systems are of key importance in the pathogenesis of depression, and should, therefore, be considered as valuable targets in treatment of patients [143]. The identification of psychiatric disease entities and their relationship with organic diseases from other medical specialties (e.g., epilepsy and Parkinson’s disease) as well as monitoring the treatment with synthetic drugs and potentially biologically active substances of plant origin can be completed with classical patient examination, electroencephalography, laboratory tests of biological samples, and population studies (including surveys). Special techniques currently include various ways of neuroimaging, mainly functional magnetic resonance imaging (fMRI) and positron emission tomography (PET), which provide information about morphological changes and functional disorders occurring in depression or characteristics of diseases accompanied by depression [144,145,146,147,148,149,150,151,152,153,154]. 

### 1.2. Phytotherapy

The treatment of depression is based on the application of a wide range of synthetic drugs, e.g., MAOIs, TCAs, and SSRIs, which cause many side effects [155,156,157,158]. Therefore, there is a search for effective bioactive compounds that can be a source of new antidepressants [159,160,161,162]. Intensive research in psychophysical herbology is being carried out to address the concerns about the safety and side effects of many synthetic antidepressants. Herbal drugs are becoming increasingly popular as an alternative to drugs prescribed for the treatment of major depressive disorders (MDD) [163,164,165,166,167,168,169].

Currently, in the search for new effective therapeutic phytochemicals for the treatment of neurological disorders, researchers demonstrate the pharmacological effectiveness of many plant species in experiments on various animal models. Active substances contained in the organs of many plant species are being tested as effective compounds in the treatment of depression. Herbs are a source of safe phytochemicals with a beneficial effect on CNS disorders [168]. The prophylaxis and adjuvant treatment of depression are based on raw materials from many plant species e.g., representatives of the genera *Aloysia* [170], *Crocus* [171,172,173,174,175], *Gladiolus* [176,177,178], *Hemerocallis* [179,180], *Hypericum* [181,182,183,184], *Lavandula* [185,186,187], *Melisa* [188,189,190], and *Valeriana* [191,192,193]. The observations described in this paper fit in the search for new antidepressants based on the pathogenetic mechanisms of depression summed up in a review by Ates-Alagoz et al. [135] and Kalkman [51].

Specific phytochemicals such as hyperforin in St. John’s wort and kaempferol or safranal in the crocus exert therapeutic effects in depressive disorders [194,195,196]. Herbal therapies are a potential alternative used in the pharmacological alleviation of depression symptoms and treatment of the disease. An additional benefit of using herbs exerting psychotropic activity is the proven lower risk of adverse effects than in the case of antidepressants that are commonly prescribed by psychiatrists [171,197,198,199]. Main neuroprotective phytochemicals are polyphenols, including flavonoids and non-flavonoids (among others phenolic acids). With their hydroxyl groups on the aromatic A and B rings and unsaturation in the C ring, flavonoids act as antioxidants scavenging reactive oxygen species (ROS) and reactive nitrogen species (RNS). The anti-inflammation activity of flavonoids is the result of antioxidant activity and modulation of signal transduction for the synthesis of proinflammatory cytokines. Phytochemicals protect mitochondrial function against mitochondrial toxicity of accumulated pathogenic amyloid beta and presynaptic protein α-synuclein (αSyn). They also increase mitochondrial biogenesis and control their quality via mitophagy, i.e., fission–fusin and cleavage of damaged mitochondria in the autophagy–lysosome system. Plant-derived compounds can directly regulate mitochondrial membrane permeabilization, i.e., the initial step in the apoptotic process. Flavonoids increase the expression of the antiapoptotic Bcl-2 protein family and prevent the mitochondrial permeability transition pore from opening. Phytochemicals change cellular signal pathways to induce the expression of neuroprotective genes—they can exhibit neurotrophic factor-like activity by binding to neurotrophic factor receptors and activating signal pathways for neuroprotection. It has been confirmed that they function as neurotrophic factors (NTFs), especially the brain-derived neurotrophic factor (BDNF) and the glial cell line-derived neurotrophic factor (GDNF), which regulate the function and survival of neurons. Polyphenols can bind to other receptors, including GABA, nicotine acetylcholine, serotonin, κ-opioid receptor, and proteins, such as monoamine oxidase, mediating survival signaling for neuroprotection. Phytochemicals activate prosurvival MAPK pathways, including PI3K/Akt and PKC, and preserve cellular function and synaptic plasticity for neuroprotection. In animal models and cultures of neuronal and glial cells, flavonoids increase the BDNF and glial cell line-derived neurotrophic factor by activation of the ERK/CREBS or PI3K/Akt pathways. Polyphenols increase tropomycin-related kinase B (TrkB) and tropomycin-related kinase A (TrkA) expression as well as neurogenesis, neuroprotection, and antidepressant activity [200].

This review compiles information about the antidepressant potential of phytochemicals contained in the organs of selected plant species from the families Iridaceae and Xanthorrhoeaceae assessed in in vivo and in vitro studies. Given the promising results of the investigations carried out by many authors, special attention was paid to antidepressants derived from representatives of the genera *Hemerocallis* and *Gladiolus*. Both these genera were selected due to their importance and common use in Ayurvedic and folk medicine. The aim of this review report is to present information concerning the antidepressant action of bioactive compounds obtained from various organs of selected species of plants from the genera *Hemerocallis* and *Gladiolus* analyzed in some animal models and clinical studies and to elucidate the mechanism of their action. The biologically active substances identified in the flowers, leaves, and roots of several *Hemerocallis* species have been classified into several groups: alkaloids, amino acid amides, amino acids, anthocyanidins, proteins, carotenoids, catechins, flavonoids, flavonols, naphthalene glycoside, glycoside, phenolic acids, lignans, naphthalene glycoside, unsaturated polyhydroxy alcohols, nucleosides, phenol derivatives, phenylpropanoids, terpenes, and vitamins (Table 1 and Table 2).

The following groups of bioactive substances have been detected in various organs of many species of the genus *Gladiolus*: alkaloids, amino acids, anthraquinones, carbohydrates, cardiac glycosides, carotenoids, chlorophyll, coumarins, nutrition elements, essential oil, fatty oil, flavonoids, hydrolysable tannins, proteins, reducing sugars, saponins, steroids, tannins, terpenoids, and vitamins. The list of biologically active substances representing these groups identified in bulbs is presented in Table 3. Substances identified in whole plants as well as aboveground parts are shown in Table 4, and those in leaves and flowers are listed in Table 5.

## 2. Methodology

This protocol is based on 200 references, including 184 original research papers and review articles as well as 12 books published by renowned publishers such as ACS Publications, American Chemical Society, American Psychiatric Association, Annual Reviews, Austin Publishers, Bentham Science Publishers, BioMed Central, BMJ Group, British Association for Psychopharmacology, Cambridge Core, Canadian College of Neuropsychopharmacology, Canadian Medical Association, Dove Medical Press, Elsevier, Hindawi Publishing Corporation, InTech, John Wiley & Sons, Karger Publishers, Lippincott Williams & Wilkins, Massachusetts Medical Society, McCraw-Hill Education, MedCrave, Medknow Publications, Molecular Diversity Preservation International, Nature Publishing Group, Oxford Academic, Polish Psychiatric Association, Public Library of Science, Routledge and Taylor & Francis, Royal Botanic Gardens, Springer, SAGE Publishing, Taylor & Francis, The Japan Medical Association, The Royal Society of London, Thieme Medical Publishers, Timber Press, Up-ToDate, Weinheim Wiley-VCH, Wiley-Blackwell, Wiley-VCH Verlag GmbH & Co. KgaA, Wiley-WCM, Wolters Kluwer Health, and Yenepoya University imprints. Besides scientific articles, two doctoral dissertations (McGill University, Montreal, Quebec, Canada, and Linköping University, Sweden) and two recent WHO reports were used in writing the paper. The literature review includes articles published before September 2019.

The literature was searched from various resources (online and offline) of the libraries at the authors’ universities. The present study underlines the promising results related to the beneficial antidepressant action of the biologically active compounds extracted from selected organs of representatives of the genus *Hemerocallis* and *Gadiolus*. Additionally, it indicates the need for further research to elucidate the mechanisms of action and to confirm the effectiveness and safety of the use of phytochemicals in the supportive treatment of depression.

## 3. Antidepressant Action of Selected Plant Species from the Genera *Hemerocallis* and *Gladiolus*

### 3.1. Hemerocallis fulva and H. citrina

The genus *Hemerocallis* belongs to the family Xanthorrhoeaceae and subfamily Hemerocallidoideae. The flowers of various *Hemerocallis* species have been used as an important ingredient in traditional Asian cuisine due to their therapeutic properties [209]. They have been applied in Chinese medicine, i.e., in the diet therapy of sleep and mood disorders [209,217]. Ethanol extracts from *Hemerocallis fulva* Linn. flowers exhibit potent antioxidant activity, which was higher in lyophilized than dried flowers [237]. The antioxidant activity of the flowers of this species has been associated with the content of caffeoylquinic acids, flavonoids, carotenoids, and anthocyanins [208]. In their studies on the biological activities of *H. fulva* L. var. *sempervirens* M. Hotta (kwanso), Taguchi and coworkers [238] demonstrated a dose-dependent scavenging action of hydroxyl radicals generated by the H_2_O_2_/UV light system in vitro. They also found a marked increase in the hepatic glutathione levels and suppression of hepatic injury induced by acetaminophen in mice orally administered with a crude acidic extract. Moreover, the kwanso extract, to some extent, inhibited the activity of cytochrome P450 3A (CYP3A), i.e., a human and homologous mice gene locus, which includes genes encoding monooxygenases catalyzing many reactions involved in drug metabolism and synthesis of cholesterol, steroids, and other lipids.

*Hemerocallis citrina* Baroni is not only used in nutrition but is also widely applied in the folk medicine of East Asia (China, Japan) and North America for improvement of emotional health. The flower and bud parts of these species are known as Wang-You-Cao in Chinese, meaning the “forget-one’s sadness” plant. The antidepressant and sedative effects of daylily flowers, commonly called the yellow flower vegetable (Huang-Hua-Cai), the golden needle vegetable (Jin-Zhen-Cai), and the Xuan-Cao flower, were mentioned in ancient medical books, including the “Compendium of Materia Medica” which is a most famous textbook [239].

Recent clinical studies have confirmed the sedative effect [240] and high efficiency of daylily flowers in mitigation of sleep and memory disorders [240,241,242,243,244]. 

A study on a group of Japanese adults showed that a two-week supplementation of diet with *Hemerocallis* flower extracts was effective in mitigation of sleep disorders and helpful in the improvement of sleep quality, daytime dysfunction, sleep disturbance, initiation and maintenance of sleep, sleepiness on rising, sleep length, and refreshment. These improvements did not persist after the following week [245]. The antidepressant-like or fatigue-relieving effects of extracts from plants of the genus *Hemerocallis* based on sleep improvement are described in detail in the study by Yoshihara et al. [246]. It was shown that alcoholic flower extracts had greater biological activity than water extracts due to the high level of phenolic compounds, including rutin, catechin, and gallic acid [217,244,247,248,249]. Rutin, i.e., a flavonoid glycoside characterized by the highest antioxidant activity, is the main component of ethanolic extracts from daylily flowers [209]. This flavonoid is mainly reported to be involved in antidepressant effects. It was proved that the application of rutin in mice resulted in a significant reduction of the immobility time in the tail suspension test (TST) as a model of depression-like behavior [250]. The tail suspension test is a simple experimental method used in scientific research for measurement of the effectiveness of antidepressants in rodents. The test is based on the observation of immobility of a rodent subjected to short-term inescapable stress.

Lin et al. [249] studied the antidepressant efficacy of ethanolic extracts from *H. fulva* flowers (DFEtoH) and rutin administered to rodents via gastric gavage and analyzed the neurotransmitter metabolism in brain regions. The authors found that the ethanolic extract of daylily flowers and rutin applied in both short- and long-time tests significantly reduced the duration of immobility in rats and increased the swimming time in the forced swim test (FST). The forced swim test is one of the most commonly used rodent behavioral assays for the evaluation of antidepressant drugs, antidepressant efficacy of compounds, and experimental manipulations aimed at rendering or preventing depressive-like states. It is based on the assumption that an animal placed in a container filled with water will first make efforts to escape but eventually will exhibit immobility, which may reflect a measure of behavioral despair. The forced swim test is a feasible stress-induced model for mimicking human-like depressive behavior and neuronal alteration. It shares some of the factors that are influenced or altered by depression in humans, including changes in food consumption, sleep abnormalities, and drug-withdrawal-induced anhedonia [251,252].

Recent neuronal studies indicate that FST enhances corticosterone excitation and leads to a reduction of newborn neuronal cells (BrdU^+^ cells) and dysregulation of neurotransmissions [249,253,254,255]. Lin et al. [249] demonstrated that, in long-term tests, daylily flowers extracts significantly increased the serotonin concentration and reduced the serotonin turnover rate in such brain regions as the hippocampus, striatum, and amygdala but not in the frontal cortex. The authors suggested that DFEtoH exerts antidepressant-like effects, possibly via regulation of the serotonergic system. They also claimed that rutin plays a very important role in the antidepressant-like effects of DFEtoH [249]. Liu et al. [256] argued that the key role in the antidepressant activity of *H. citrina* ethanolic extracts (HCE) is played by their anti-inflammatory properties, which at least partially restore or improve the function of monoaminergic and neurotrophin systems. Upon chronic HCE administration, the authors demonstrated enhanced levels of monoamines and the brain-derived neurotrophic factor (BDNF) in a rodent depression-like model together with increased sucrose preference in the sucrose preference test (SPT). Moreover, HCE inhibited the expression of interleukin-1 beta (IL-1β), interleukin-6 (IL-6), and tumor necrosis factor-alpha (TNF-α) and reduced the indoleamine 2,3-dioxygenase (IDO) activity in the frontal cortex and hippocampus in rats exposed to chronic unpredictable mild stress (CUMS) [256].

The efficacy of ethanolic extracts from *H. citrina* flowers (HCE) in the reversal of behavioral disorders and monoamine transmitter dysfunction in stressed rats was demonstrated by Yi et al. [257]. The results of these experiments indicate that the administration of HCE (65 and 130 mg/kg) eliminated anhedonia symptoms, which was manifested by increased interest in reward stimuli in the SPT test and increased activity in the FST test. Moreover, it was confirmed that the HCE-produced antidepressant-like effect in a corticosterone-induced depression-like model of rats was at least partly mediated by the brain-derived neurotrophic factor and its protein receptor (BDNF-TrkB) signaling in the frontal cortex and hippocampus region [257]. As demonstrated by Li et al. [258], ethanol extracts from *H. citrina* Baroni (HCE) administered orally to mice exhibit antidepressant and anti-inflammatory effects as a result of inhibition of the nuclear transcription NF kappa B pathway (NF-κB; nuclear factor kappa B). NF-κB is present in the nuclei of B lymphocytes. It plays an essential role in inflammation and immune processes. It inhibits and induces programmed cell death (apoptosis), enhances the formation of new thin-walled capillaries from already existing ones (angiogenesis), and accelerates tissue growth and cell proliferation [258,259].

In studies conducted in a lipopolysaccharide-induced murine model of depression, it was demonstrated that lipopolysaccharides (LPS) activated the NF-κB factor in the prefrontal cortex and induced expression of nitric oxide synthase (iNOS) and cyclooxygenase-2 (COX-2); these activities were normalized after prior administration of HCE. Additionally, HCE was shown to reverse significantly the reduction of sucrose preference with LPS [259]. Strong evidence was provided for the complete toxicological safety of oral administration of active HCE extracts. The effectiveness of hydroalcoholic extracts from *H. citrina* flowers in mitigation of depression symptoms was also underlined by Du et al. [180] in their study of mouse males in behavioral models (tail suspension tests TST and open field tests OFT). It was found that the antidepressant-like effects of hydroalcoholic *H. citrina* extracts, especially those prepared using 75% ethanol (i.e., HCE75), were mainly related to the presence of rutin and hesperidin flavonoids.

Studies of the antidepressant mechanism of hesperidin demonstrated that the up-regulation of BDNF induced by this flavonoid was mediated in an extracellular signal-regulated kinase (ERK)-dependent manner. Hesperidin reversed the elevation of the immobility time and the reduction of sucrose preference in mice induced by chronic mild stress. Moreover, hesperidin treatment ameliorated the increase in serum corticosterone levels and the decrease in hippocampal ERK phosphorylation and BDNF levels in mice exposed to mild chronic stress [260].

Similarly, Zhai et al. [261] postulated that the antidepressant activity of flavonoids extracted from daylily flowers was associated with the involvement of brain monoamine neurotransmitters. It was revealed that the main role in the induction of the antidepressant activity of HCE extracts was played by the serotonergic and dopaminergic systems. Correspondingly, the results of investigations conducted by Gu et al. [179] on mice indicate the involvement of the monoaminergic system in the mechanism of alleviation of depression symptoms with the use of HCE. The authors postulated that the antidepressant activity of HCE was associated with the serotonergic (5-HT(1A) and 5-HT(2) receptors), noradrenergic (α(1)-, α(2)-, and β-adrenoceptors), and dopaminergic (D(2) receptor) systems as well as the elevation of monoamine neurotransmitters (5-HT, NA, and DA) levels in mouse brain. It was revealed that HCE enhanced the 5-HT and NA levels in the frontal cortex and hippocampus, and elevated the DA levels in the frontal cortex. Furthermore, oral administration of HCE (90, 180, and 360 mg·kg^−1^) significantly limited the immobility time in both the forced swim test (FST) and the tail suspension test TST without changes in the locomotor activity evaluated in the open-field test (OFT). The antidepressant-like effect of HCE (360 mg·kg^−1^, p.o.) in the TST was not found after pretreatment with a 5-HT(1A) receptor antagonist WAY 100635 (0.1 mg·kg^−1^, administered subcutaneously-s.c.), a 5-HT(2) receptor antagonist cyproheptadine (3 mg·kg^−1^, i.p.), an α(1)-adrenoceptor antagonist prazosin (62.5 μg·kg^−1^, i.p.), an α(2)-adrenoceptor antagonist yohimbine (1 mg·kg^−1^, i.p.), a β-adrenoceptor antagonist propranolol (5 mg·kg^−1^, i.p.), or a dopamine D(2) receptor antagonist sulpiride (50 mg·kg^−1^, i.p.), and was not recorded for a dopamine D(1) receptor antagonist SCH23390 (0.05 mg·kg^−1^, s.c.). It is believed that the total phenolic extract of *H. citrina* (HCPE) contains the main active ingredients, which have an emotion improvement effect. Xu et al. [262] reported that HCPE treatment via gastric gavage, especially with a dose of 40 mg/kg/day, effectively improved the emotions and cognition-related behavior in depression in rats induced with chronic unpredictable mild stress (CUMS) procedures. The authors evaluated the antidepressant effect of HCPE with the sucrose preference test, open field test, and body weight, while the improvement of cognitive processes was investigated with the Morris water navigation task, also known as the Morris water maze (MWM) test. It was revealed that the mechanism of the positive action of HCPE on the CUMS rats was associated with the regulation of monoamine neurotransmitters (5-HT, DA, and NA) and brain-derived neurotropic factor (BDNF) levels in the brain and with the alleviation of the corticosterone (CORT) level. Moreover, it was found that HCPE reduced the malondialdehyde (MDA) level in the frontal cortex of model rats. This shows inhibition in the rate of lipid peroxidation and alleviation of oxidative stress.

As demonstrated by Zhang et al. [215], *H. fulva* leaves contain compounds with strong antioxidant activity. They include roseoside, phlomuroside, lariciresinol, quercetin 3-O- β -d-glucoside, quercetin 3,7-O- β -D-diglucopyranoside, quercetin 3-O- α -L-rhamnopyransol-(1→6)- β -D-glucopyranosol-7-O- β -D-glucopyranoside, isorhamnetin-3-O- β -D-6′-acetylglucopyranoside, and isorhamnetin-3-O- β -D-6′-acetylgalactopyranoside.

The results of investigations conducted by Tian et al. [239] on a PC12 cell line derived from a pheochromocytoma of rat adrenal glands showed that phenolic acid derivatives (0.59% *w/w* in the flowers) and flavonoids (0.60% *w/w*) were the most biologically active components of hydroalcoholic daylily (*H. citrina*) extracts. Both these groups of compounds were characterized by a highly similar level of neuroprotection but had different effects on the release of neurotransmitters. The presence of phenolic acid derivatives in corticosterone- and glutamate-treated PC12 cells resulted in an increased (DA) level in the cell culture medium, whereas flavonoids elevated the ACh and 5-HT levels. A brief summary of the results of studies on the antidepressant effect of *Hemerocallis* sp. is presented in Table 6.

### 3.2. Gladiolus Dalenii

*Gladiolus dalenii* Van Geel (family Iridaceae) is one of the most widely distributed species of genus *Gladiolus*, ranging from eastern South Africa and Madagascar through tropical Africa and into western Arabia. The bulbs and corms of this ornamental erect herb were used in ethnomedicine, especially in Cameroon, as a cure for various ailments, including some central nervous system disorders such as epilepsy, convulsions, schizophrenia, and mood disorders [263]. In the local Babadjou language spoken in the western region of Cameroon, this species is called “Mantsap Letoupuh”, which means “wild onion” [264,265]. The anticonvulsant and sedative effects of *G. dalenii* extracts studies in two in vivo mouse models (maximal electroshock MES and pentylenetetrazol PTZ-induced convulsions) have recently been confirmed by Ngoupaye et al. [177]. In their investigations, the authors demonstrated a very high efficacy of macerated aqueous and lyophilized extracts of *G. daleni* against PTZ- and MES-induced seizures, i.e., 100% (PTZ) and 83% (MES), respectively. Co-administration of *G. dalenii* with diazepam resulted in an additive effect, in contrast to co-administration thereof with a selective benzodiazepine receptor antagonist flumazenil or the GABAergic antagonist FG-7142. It was also found that the sedative activity of the *G. dalenii* macerate was manifested in a reduction of the latency time to sleep and an increase in the total duration of diazepam-induced sleep by approximately two hours.

Although depression (affective disorder) and epilepsy are widely recognized as completely separate disease entities whose diagnostics and treatment pertain to different medical specialties, it has been suggested that there are grounds for identification of many common elements, i.e., similarities between these diseases in their bioelectrical background or phenomena accompanying these two diseases [266]. It has been documented that epilepsy patients suffer from depression more often. It is estimated that around 35% of them develop depression at the same time. The possible causes of depression in patients with epilepsy include: (i) seizures, (ii) hormones, (iii) side effects produced by medications, and (iv) psychological factors. Seizures and disease-related changes with varying severity in epilepsy can lead to mood disorders, including depression. Hormone levels, especially sex hormones, affect the mood and brain function, thereby increasing the risk of development of both epilepsy and depression. This effect is more pronounced in women than in men. Anti-seizure drugs and medications, especially barbiturates, affect the mood centers in the brain, raising the risk of depression. The negative emotions associated with such a long-term disease as epilepsy and its troublesome symptoms can trigger negative emotions, e.g., sadness, anxiety, embarrassment, or anger, which can lead to depression. The “burden of epilepsy” (epilepsy-related stress) explains depression in many patients, but acute and temporary seizure-related states of depression or suicidality have also been reported. Evidence has been found that seizures and mood disorders, including depression, may share the same genetic cause in some epilepsy patients. It is believed that modern antidepressants (SSRI, SNRI, noradrenergic, and specific serotonergic antidepressant—NaSSA) can be safely used in epilepsy; however, due to the lack of relevant studies, the evidence is still incomplete, especially in the case of mild depression [257,258,259,260,261,262,263,264,265,266,267,268,269,270,271]. The current knowledge of the biochemical and structural background of depression is wide, and the issue has been sufficiently recognized. In turn, the relationships between depression (affective disorder) and bioelectrical phenomena in the brain (EEG activity) remain unexplained. Certain bioelectrical phenomena accompanying the different forms of antidepressant therapy (e.g., changes in the threshold level) are recognized as accidental side effects or effects without clinical importance rather than a possible mechanism of action or a biological mechanism of the etiopathogenesis of depression. Additionally, different forms of therapy have different effects on the EEG function, although they result in improvement or stabilization of the disease-suppressed mood [266].

It has been evidenced that *G. dalenii* lyophilizates ameliorate scopolamine-induced amnesia in male rats through inhibition of oxidative stress in the brain and enhancement of cholinergic neurotransmission. *Gladiolus dalenii* was found to reduce acetylcholinesterase activity in the hippocampus and prefrontal cortex. It also decreased the level of malondialdehyde and increased the level of glutathione in the hippocampus of scopolamine-treated rodents. This effect was accompanied by *the* reversion of memory dysfunction in the Morris water maze, novel object location, and recognition tasks [272].

Ngoupaye et al. [176] showed antidepressant-like effects of the aqueous macerate of the bulb of *Gladiolus dalenii* Van Geel in a rat model of epilepsy-associated depression induced by combined administration of atropine and pilocarpine. This effect was manifested by significantly reduced immobility times in the forced swim test (FST) and the locomotor activity assessed in the open field test (OFT). The authors claimed that the antidepressant activity of *G. dalenii* is mediated by the restoration of the activity of the hypothalamic-pituitary-adrenal (HPA) axis, as a reduced level of plasma corticosterone (CORT) and adrenocorticotropic hormone (ACTH) rather than the adrenal gland weight was shown. Moreover, the elevation of another depression-related parameter i.e., an increase in the hippocampal levels of brain-derived neurotrophic factor (BDNF), was observed. The antidepressant-like properties of *G. dalenii* in epilepsy-associated depressive states were comparable or even higher to those of fluoxetine, which is an SSRI used frequently to treat depression.

Similarly, other results reported by these authors indicate that aqueous extracts from the *G. dalenii* corm applied per o.s. had an antidepressant effect in mice, which was assessed in common experimental models of depression, namely OFT, FST, and TST [178]. This effect appeared to be even stronger than that of the common antidepressants imipramine and fluoxetine as well as the N-methyl-D-aspartate (NMDA) receptor antagonist D-(-)-2-amino-7-phosphonoheptanoic acid (D-AP7). The authors suggest that the antidepressant properties of *G. dalenii* are mediated through interactions with NMDA and the serotonergic and/or noradrenergic systems. Studies aimed at investigating the mechanism of action showed that *G. dalenii* extracts significantly antagonized the effect of NMDA. *Gladiolus dalenii* extracts in combination with NMDA, reuptake inhibitor fluoxatine, and multitarget antidepressant imipramine markedly reduced the immobility time in rodents. It was also found that neither the *G. dalenii* extract alone nor its combinations with the NMDA ligands, imipramine, and fluoxetine enhanced spontaneous locomotor activity in mice.

In turn, based on the elevated plus maze (EPM) test, measurements of the stress markers, and reproductive parameters, Fotsing et al. [273] postulated that orally applied aqueous extracts of *G. dalenii* protect from stress-induced behavioral, neurochemical, and reproductive changes in female albino rats. *Gladiolus dalenii* extracts markedly increased the number of entries and the time spent in the open arm exploration of the EPM. The chronic immobilization stress-induced elevated corticosterone, progesterone, and prolactin levels were antagonized by the application of the *G. dalenii* extract. Moreover, the drop in the reproductive hormones (follicle-stimulating hormone, luteinizing hormone, glucose estradiol), as well as the changes in the estrous cycle duration (triglycerides, cholesterol) and in the level of neurotransmitters (serotonin, adrenaline) caused by the chronic immobilization stress, were normalized in the *G. dalenii*-treated rats. It is assumed that the beneficial effects could be related to the bioactive molecules and secondary metabolites such as alkaloids and flavonoids contained in the plant. However, detailed investigations focused on identification and characterization of their activity and mechanisms of action are still needed. A brief summary of the results of studies on the antidepressant effect of *Gladiolus* sp. is presented in Table 7.

### 3.3. Synergistic Activity of Phytocompounds 

The natural co-occurrence of the above-mentioned substances in the described herbs gives hope for a stronger therapeutic effect than the same substances tested separately. Further studies can focus on the probable synergistic activity of plant compounds, i.e., when their common effect on the human organism is greater than the sum of effects caused by each of them individually. Many biologically active substances that are commonly found in plant products exert a positive effect on treatment, including the neuroprotective, antioxidant, and anti-inflammatory effects of phytochemicals. This is especially important in depression, as oxidative stress and inflammation are recognized as a significant factor involved in the pathogenesis of this disorder, especially the major depressive disorder and various other nervous system disorders [274,275,276]. There is strong evidence that inflammation and oxidative stress are the main contributors to the progression that occurs in major depressive disorder. Depressed patients show elevated levels of inflammatory biomarkers and markers of oxidative damage to biomolecules (lipids, proteins, and DNA) as well as low levels of antioxidants such as co-enzyme Q-10, glutathione, ascorbic acid, vitamin E, and polyunsaturated fatty acids. The synergistic action of an activated immune–inflammatory system and increased oxidative stress impedes the elucidation of depression pathogenesis. It has been evidenced that antidepressants decrease oxidative stress in animal models of chronic stress and depressed patients and simultaneously noticeably improve the buffering mechanisms of inflammation processes [277,278]. Oxidative stress is a result of the loss of biological balance between reactive oxygen species (ROS) and antioxidants, leading to alterations in biomolecules and loss of control of intracellular redox-related signaling pathways. Reactive oxygen species not only act as pivotal secondary messengers in signal transduction but also significantly affect inflammatory pathways by activating nuclear factor-*κ*B and mitogen-activated protein kinase family of stress kinases. The excess of ROS inflicts damage to cellular constituents with the formation of pro-inflammatory molecules, such as malondialdehyde, 4-hydroxynonenal, neoepitopes, and damage-associated molecular patterns promoting immune response, ultimately leading to cell death. The failure of cells to adapt to the changes in redox homeostasis and the subsequent cell death, together with the damage caused by inflammatory mediators, are postulated as causes of progression of the disorder [279]. The cascade of antioxidant and inflammatory events is administered via various transcription factors, with a special role in depression played by nuclear factor (erythroid-derived 2)-like 2 (Nrf2) and nuclear factor-*κ*B (NF-*κ*B). However, the molecular mechanisms through which impaired redox homeostasis and neuroinflammation affect the neuronal environment and contribute to depression are continually discussed [274,280,281,282,283,284].

There are few papers describing the synergistic effects of many groups of bioactive compounds, including those exhibiting antidepressant effects and occurring in various organs of *Hemerocallis* and *Gladious*. For example, in terms of anti-inflammatory and antioxidant activity, synergistic effects have been shown between polyphenol and polysaccharide fractions, especially when these compounds are used in combination with triterpenic acids. In terms of antimicrobial activity, phenolic acids have been found to enhance the effects of flavonoid sub-classes [285,286,287,288]. The issue of the synergistic antioxidant activity of natural products, including the synergistic interactions between the antioxidant components of various natural products, synergism between antioxidant components of different herbs, and synergism between synthetic antioxidants and natural products, is described in detail in the paper by Sonam and Guleria [289]. Further extensive research into the efficacy of mechanisms of phytocompounds is needed to design and develop highly effective novel natural or combined medicines. This is not easy due to the complex nature of the plant extracts.

Herbs often interact with drugs, thus triggering serious reactions. Herbs and herbal drugs usually contain several bioactive compounds, which largely increases the likelihood of interactions taking place after administration thereof. In turn, synthetic formulations usually contain a single chemical substance; therefore, the likelihood of herb–drug interactions is theoretically substantially higher than drug–drug interactions. The herb–herb interactions are even more complex. Based on the nature of the interactions, two broad types of synergy can be distinguished: pharmacodynamic and pharmacokinetic. The former type of synergy is noted between two drugs directed at the similar receptor target or physiological system. In turn, the latter results from the processes of drug absorption, distribution, biotransformation, or elimination [290,291]. This issue should be explored more profoundly in the case of herbs with more than one type of biologically active substance and especially in the case of herbal blends.

## 4. Conclusions

The flowers and buds of *Hemerocallis citrina* Baroni and *H. fulva* L., as well as the bulbs and corms of Gladiolus dalenii Van Geel, are used in ethnomedicine as a cure for various ailments, including some central nervous system disorders. Given their properties, they have been commonly used for enhancement of emotional state and alleviation of sleep and mood disorders. Recent clinical studies have confirmed the antidepressant activity of daylily flowers and *G. dalenii* bulbs and corms. They have been shown to have sedative activity and to be effective in the mitigation of sleep and memory disorders as well as the elimination of anhedonia symptoms. This beneficial antidepressant effect of mainly alcoholic, and to a lesser extent, aqueous extracts from the daylily manifested by improvement of the mood and alleviation of depressive symptoms is attributed to the high level of antioxidants contained therein, e.g., carotenoids, flavonoids (hesperidin, catechin, and gallic acid), and anthocyanins, but primarily to a compound from the group of flavonoid glycosides, i.e., rutin. In turn, it is assumed that the beneficial effects of the *Gladiolus* species are related to such bioactive molecules and secondary metabolites as alkaloids and flavonoids. The following mechanisms of the antidepressant activity of daylily extracts have been proposed: (1) inhibition of the reuptake of monoamine neurotransmitters (DA, NA, 5-HT), (2) improvement of BDNF, and (3) NMDA receptor antagonism. The latter two mechanisms are also postulated to underlie the antidepressant action of gladiolus extracts. Moreover, the antidepressant activity of *G. dalenii* is mediated by GABA-α agonism. The antidepressant activity of *G. dalenii* could also be mediated by the restoration of the activity of the HPA axis, as indicated by the reduced level of plasma CORT and ACTH but not the adrenal gland weight. Literature data provide strong evidence that the antidepressant effect of *Hemerocallis* and *Gladiolus* sp. results from the anti-inflammatory and antioxidant properties of the bioactive compounds contained therein. The anti-inflammatory effect is a consequence of inhibition of the NF-κB and inhibition of the expression of pro-inflammatory interleukins 1 and 6. In turn, the antioxidant activity mitigates the negative effects of oxidative stress. Extracts from *Hemerocallis* sp. and *Gladiolus* sp. may be a potential safe (without side effects) and effective drug for depression and its associated cognitive deficit. Promising results for *Hemerocallis* and *Gladiolus* extracts as adjunctive therapy in the treatment of depression in combination with pharmacological therapy have been achieved. However, there is still a need for detailed investigations focused on the identification and characterization of the active compounds and their mechanism of action as well as their activity depending on the cause of depression and the occurrence of associated disorders.

## Figures and Tables

**Table 1:** Bioactive compounds in flowers of several species of the genus *Hemerocallis.*

Group of Bioactive Compounds	Bioactive Compounds	Species	Author
**Flower**			
**Alkaloids**	hemerocallisamine I–VII	*Hemerocallis* sp.	[201,202]
	2-formylopyrole hemerokallisamine I	*H. fulva* L. *H. flava* L. *H. minor* Mill.	[203]
**Anthocyanidins**	cyanidin3-rutinoside; delphinidin-3-rutinoside	*H. fulva* L.	[204]
	cyanidin; delphinidin; pelargonidin; peonidin; petunidin	*Hemerocallis* sp.	[205]
**Amino acids**	tryptophan derivative; tyrosine	*H. fulva* L.	[206,207,208]
Amino acid amides	longitubanine a		[209]
**Protein**	globulins		[206,207]
**Carotenoids**	lutein, zeaxanthin; lutein; lutein-5,6-epoxide; neoxanthin; trans-β-carotene; violaxanthin; violeoxanthin; β-cryptoxanthin; zeaxanthin	*H. disticha* Donn	[210,211]
	β-karoten, lutein; zeaxanthin	*H. fulva* L.	[204,212]
	carotene; lycopene	*Hemerocallis* sp.	[205]
**Flavonoids**	agipenin; kaempferol; luteolin; myricetin; quercetin; rutin		[205,213]
	hesperidin; hyperoside; isoquercitrin; isorhamnetin 3-o-glucoside; kaempferol 3-rutinoside; kaempferol-3-o-galactoside; quercetin 3,7-o-β-d-diglucopyranoside; quercetin 3-o-β-d-xylopyranoside; rutin	*H. citrina* Baron	[214]
	chrysin; chrysoeriol 7-o-[β-d-glucuronopyranosyl(1→2)(2-o-trans-feruloyl)-β-d-glucuronopyranoside; hesperidin; isorhamnetin 3-o-glycosides; isorhamnetin-3-o-β-d-6′-acetylglucopyranoside; kaempferol 3-o-{α-l-rhamnopyranosyl(1→6)[α-l-rhamnopyranosyl(1→2)]}-β-d- galactopyranoside; kaempherol; myricetin; naringenin; naringin; n-butyl 4-trans-o-caffeoylquinate; pinocembrin; quercetin 3,7-o-β-d-diglucopyranoside; quercetin 3-o-α-l-rhamnopyransol-(1→6)-β-d-glucopyranosol-7-o-β-d-glucopyranoside; quercetin 3-o-β-d-glucoside; quercetin; rutin	*H. fulva* L.	[208,209,215,216]
**Glycosides**	orcinol beta-d-glucopyranoside; phenethyl β-d-glucopyranoside; phloretin 2′-o-β-d-glucopyranoside; phloretin 2′-o-β-d-xylopyranosyl-(1→6)-β-d- glucopyranoside		[209]
**Phenolic acids**	caffeoylquinic acid; gallic acid		[208,217]
	4-o-p-coumaroylquinic acid; gallic acid	*H. citrina* Baron	[214]
**Naphthalene glycosides**	stelladerol	*H. fulva* L.	[208,209]
**Unsaturated polyhydroxy alcohols**	ascorbic acid		[212]
**Nucleosides**	adenosine; guanosine		[208]
**Phenol derivatives**	hemeratrol a	*H. minor* Mill.	[218]
**Phenylpropanoids**	4-o-caffeoylquinic acid; caffeic acid; chlorogenic acid	*H. citrina* Baron	[214]
**Terpenes**	hemerolides a–c	*H. minor* Mill.	[218]

**Table 2:** Bioactive compounds in leaves and roots of several species of the genus *Hemerocallis.*

Group of Bioactive Compounds	Bioactive Compounds	Species	Author
**Leaves**			
**Amino acid amides**	pinnatanine	*H. fulva* L.	[209,215]
**Catechins**	catechin	*Hemerocallis* sp.	[213]
**Glucoside**	phlomuroside	*H. fulva* L.	[209,215]
**Terpenoids**	roseoside		
**Lignans**	lariciresinol		[209,215]
**Nucleosides**	adenosine		[209,215]
**Phenylpropanoids**	chlorogenic acid	*Hemerocallis* sp.	[213]
**roots**			
**Alkaloids**	hemerominory A-H; γ-lactam	*H. minor* Mill	[219]
**Anthraquinones**	2-hydroksychrysophanol; kwanzoquinones A, B, C, D, E, F, G; rhein	*H. fulva* L.	[220]
**Flavonols**	6-methylluteolin		
**Naphtalene glycosides**	5-hydroxydianellin; dianelin		
**Vitamins**	α-tocopherol		

**Table 3:** Bioactive compounds in bulbs of several species of the genus *Gladiolus.*

Group of Bioactive Compounds	Bioactive Compounds	Species	Author
**Anthraquinones**	methyl trans-p-methoxycinnamate; methyl 8-hydroxy-3,6,7-trimethoxy-1-methylanthraquinone-2-carboxylate (gandavensin B); methyl 8-hydroxy-3,6-dimethoxy-1-methylanthraquinone-2-carboxylate; methyl 8-hydroxy-3-methoxy-6,7-methylenedioxy-1-methylanthraquinone-2-carboxylate (gandavensin A); 5,7-dimethoxy-2-methylchromone; 5-hydroxy-2-hydroxymethyl-7-methoxychromone	*G. gandavensis* Van Houtt.	[221]
	deoxy-erythrolaccin; laccaic acid D methylester; physcion	*G. segetum* Ker-Gawl.	[222]
	1,6,7-trihydroxy-3-methoxy-8-methyl-anthraquinone; 1-hydroxy-3,6,7-trimethoxy-8-methyl-anthraquinone	*G. psittascinus* Hook	[223]
**Cytokinins**	isopentenyl adenine; zeatin	*G. grandiflorus* L.	[224]
**Steroids**	(–)-dehydrodiconiferyl alcohol; (+)-demethoxypinoresinol; (+)-pinoresinol monomethylether; (+)-pinoresinol; 6′-Opalmitoyl-3-O-sitosterol glucoside; neolignan; β-sitosterol-3-O-glucoside	*G. segetum* Ker-Gawl.	[222]
**Terpenes**	2β, 3β, 16α, 28-tetrahydroxy-olean-12-ene-23-oic acid; medicagenic acid		[225]

**Table 4:** Bioactive compounds in aerial parts of several species of the genus *Gladiolus.*

Group of Bioactive Compounds	Species		Author
**Whole Plant**			
**Anthraquinones**	emodin	*G. atroviolaceus* Boiss	[226]
**Flavonoids**	kampferol-3-o-rhamnoside; kampferol-3-o- β-glucopyranoside; quercetin-3-o-rhanmnoside		
**Phytosterols**	stigmasterol glucoside		
**Terpenoids**	gladioloic acid A; gladioloic acid B		
**Aerial parts**			
**Anthraquinones**	1-hydroxy-3,6,7-trimethoxy8-methylanthraquinone; 3,8-dihydroxy-4,7-dimethoxy-1-methylanthraquinone2-carboxylic acid methyl ester; 3,8-dihydroxy-6-methoxy-1-methylanthraquinone-2-carboxylic acid; 3,8-dimethoxy-1-methylanthraquinone-2-carboxylic acid methyl ester; desoxyerythrolaccin; methyl 3-methoxy-1-methyl-9; 10-dioxo-8-(beta-d-glucopyranosyloxy)-9,10-dihydroanthracene-2-carboxylate; methyl 8-hydroxy-4,7-dimethoxy-1-methyl-9,10-dioxo-3-(beta-d-glucopyranosyloxy)-9,10-dihydroanthracene-2-carboxylate	*G. segetum* Ker-Gawl	[227,228,229,230]
**Flavonoids**	apigenin-7-O-alpha-L-rhamnoside; astragalin-2”-O-beta-D-glucopyranoside kaempferol; glycerol-alpha-monohexacosanate; nicotiflorin; quercetin-3-O-(6”-O-Ecaffeoyl)-beta-D-glucopyranoside; tamarixetin-3-robinobioside	*G. gandavensis* Van Houtt.	[231]
	2, 5, 6- trihydroxy-2, 4-dimethyl-6-metoxy-1-benzofuran-3-one; kaempferol-3-O-β-D-glucopyranoside8; quercetin-3-O-β-D-glucopyranoside8	*G. segetum* Ker-Gawl	[229,230]
**Phytosterols**	β-sitosterol, daucosterol	*G. gandavensis* Van Houtt.	[232]
	ergosterol, stigmasterol	*G. segetum* Ker-Gawl	[229]
**Terpenoids**	29-o-(β-d-glucopyranosyl)-2β,3βdihydroxyolean-12-en-28-oic acid; 3-o-(β-d-xylopyranosyl)-29-o-(β-d-glucopyranosyl)-12-en-28-oic acid; β-d-glucopyranosyl] ester	*G. gandavensis* Van Houtt	[232]
	betulinic acid	*G. segetum* Ker-Gawl	[229]
**Fatty acyl glycosides of mono- and disaccharides**	isopentyl gentiobioside	*G. gandavensis* Van Houtt.	[231]
**Sterol lipoprotein**	cholesterol	*G. segetum* Ker-Gawl	[229]
**Nucleosides**	adenosine	*G. atroviolaceus* Boiss.	[226]

**Table 5:** Bioactive compounds in leaves and flowers of several species of the genus *Gladiolus.*

Group of Bioactive Compounds	Bioactive Compounds	Species	Author
**Leaf**			
**Anthocyanins**	cyaniding; delphinidin; malvidin; pelargonidin	*Gladiolus “*Green Star”, “Red Flair”, “Pink Event”, “Violetta”, “Ice Cap”	[233]
**Flower**			
**Flavonoids**	flavonol glycosides; kaempferol; kaempferol 3-o-rutinoside; kaempferol 3-o-sophoroside; laricitrin; myricetin; quercetin; quercetin 3-o-rutinoside; syringetin	*G. grandiflora* “Ariake”	[234]
**Anthocyanins**	cyaniding; delphinidin; malvidin; pelargonidin; peonidin; petunidin	*Gladiolus sp.*	[235]
	malvidin 3,5-di-o-glucoside (malvin); malvidin glycosides	*G. grandiflora* “Ariake”	[234]
	3,5-di-o-glucosides of petunidin; 3-o-rutinoside-5-oglucosides of cyaniding; cyaniding; malvidin; malvidin 3-o-glucoside, pelargonidin 3-o-rutinoside; pelargonidin; peonidin	*Gladiolus* of 18 cultivars	[236]

**Table 6:** A brief summary of the results of studies on the antidepressant effect of *Hemerocallis flava* L. and *H. citrine* Baroni (Xanthorrhoeaceae).

Plant Organ	Extract, Active Compound	Dosage and the Way of Administration/Biological Object	Main Results	Proposed Mechanism of Antidepressant Action	Author
***Hemerocallis citrina* Baroni**					
**Flower**	ethanol extract	90, 180 or 360 mg·kg^−1^, p.o./*	Reduced immobility time in FST and TST. Enhanced 5-HT and NA levels in the frontal cortex and hippocampus. Elevated DA levels in the frontal cortex	Via the serotonergic (5-HT_1A_ and 5-HT_2_ receptors), noradrenergic (α1-, α2- and β-adrenoceptors) and dopaminergic (D-2 receptor) systems	[179]
**Flower**	hydroalcoholic extracts, flavonoids – rutin, hesperidin	400 mg·kg^−1^, p.o./*	Reduced immobility time in TST and improvement of locomotor activity in OFT. Increase in the serotonin and dopamine levels in the central nervous system	Via the serotoninergic and dopaminergic systems. The presence of flavonoids with sub-additive interaction between rutin and hesperidin	[180]
**Flower**	phenolic (phenolic acid derivatives, flavonoids) and non-phenolic fractions of the hydroalcoholic extract	24 h pretreatment with fractions 0.3–5.0 mg raw material/mL /***	Neuroprotective effects against corticosterone and glutamate-induced damage in PC12 cells exerted by phenolics, but not non-phenolic fractions. Similar extent of the neuroprotective effect of phenolic acid derivatives and flavonoids, but quite different release of neurotransmitters	Regulation of neurotransmitters. Influence of phenolic acid derivatives on the release of dopamine DA and NA. Modulation of the release of 5-HT, NA, and ACh by flavonoids	[239]
**Flower**	ethanol extract	130 mg kg^−1^ for four weeks via gavage/**	Amelioration of CUMS-induced depressive symptoms. Reversion of the decreased sucrose preference in SPT, inhibition of IL-1β, IL-6, and TNF-α expression, as well as IDO activity in the frontal cortex and hippocampus	Restoration or improvement of monoaminergic and neurotrophin systems due to the anti-inflammatory properties of daily flower extracts	[256]
**Flower**	ethanol extract	32.5; 65 or 130 mg·kg^−1^ BW, p.o./**	Reversion of the corticosterone induced (40 mg/kg, s.c.) depression-like behaviors in SPT and FST	Via BDNF-TrkB (brain-derived neurotrophic factor and its receptor) signaling in the frontal cortex and hippocampus	[257]
**Flower**	ethanol extract	180, 360, and 720 mg·kg^−1^ per eight weeks, p.o./*	Decreased total cholesterol levels without any significant histopathological changes in the liver and kidney. Reversion of the reduction of sucrose preference (SPT) with LPS. Normalization of NF-κB activation as well as the expression of iNOS and COX-2 in an LPS-induced depressive-like model	Inhibition of the NF-κB signaling pathway in the prefrontal cortex	[258]
**Flower**	total phenols extract	10, 20, and 40 mg·kg^−1^ daily, via gastric gavage **	Improvement of depression-like emotional status, amelioration of depression-related behavior in TST, and association of cognitive deficits in MWM induced by chronic unpredictable mild stress (CUMS) procedures due to HCPE, especially at 40 mg kg^−1^	Regulation of neurotransmitters (5-HT, DA, and NE) and BDNF levels in the brain. Reduced CORT level in the serum. Alleviation of oxidative stress manifested by decreased MDA in the frontal cortex	[262]
***Hemerocallis fulva*** **L.**					
**Flower**	ethanol extract, flavonoid rutin	3, 15, or 30 g·kg^−1^ BW for one or two weeks via oral gavage/**	Reduced immobility time and increased swimming time in FST. Increase in the serotonin, norepinephrine, and dopamine levels in the frontal cortex, hippocampus, striatum, and amygdala. DFEtoH elevated the serotonin level and reduced the serotonin turnover rate in these brain regions but not in the frontal cortex.	Regulation of the serotonergic system. Role of rutin in the antidepressant-like effects of DFEtoH through blockage of MAO and elevation of the synaptic neurotransmitter level	[249]

Explanations: biological object: * - mice, ** - rats, *** - rat pheochromocytoma cells (PC12); 5-HT—serotonin; ACh—acetycholine; BDNF—brain-derived neurotropic factor; CORT—corticosterone; COX-2—cyclooxygenase-2; CUMS—chronic unpredictable mild stress; DA—dopamine; DFEtoH—ethanol extract of daylily flowers; FST—forced swim test; HCE—ethanol extract of *H. citrina*; HCPE—total phenolic extract of *H. citrina*; i.p.—intraperitoneally; IDO—indoleamine-2,3-dioxygenase; IL-1β—interleukin - 1 beta; IL-6—interleukin-6; iNOS—inducible nitric oxide synthase; kBW—body weight; LPS—lipopolysaccharide; MAO—monoamine oxidase; MDA—malondialdehyde; MWM—Morris water maze test; NA—noradrenaline; NF-κB—nuclear factor-κB; OFT—open field test; p.o.—per os, administered orally; SPT—sucrose preference test; TNF-α—tumor necrosis factor-alpha; TST—tail suspension test.

**Table 7:** A brief summary of the results of studies on the antidepressant effect of *Gladiolus dalenii* Van Geel (Iridaceae).

Plant Organ	Extract, Active Compound	Dosage and the Way of Administration/Biological Object	Main Results	Proposed Mechanism of Antidepressant Action	Author
**Corm or bulbs**	aqueous extract	15 mg·kg^−1^ for 7 days, i.p./**	Counteraction of associated depressive states induced with pilocarpine combined with atropine pretreatment. Reduction of the immobility time assessed in FST and enhancement of spontaneous locomotor activity in OFT. Drop in the levels of ACTH, CORT, but not the adrenal gland weight. Increase in the level of BDNF in the hippocampus	Restoration of the activity of the HPA axis and an increase in the BDNF level in the hippocampus	[176]
		7.5; 15 and 150 mg kg^−1^, p.o./*	Reduction of the immobility time in FST and TST. Antagonization of the effect of N-methyl-D-aspartate (NMDA) after administration of the moderate and highest doses of the extract. Shortening of the immobility time at the sub-effective dose (7.5 mg kg^−1^) in combination with either D-(−)-2-amino-7-phosphonohepta- noic acid (D-AP7) (the NMDA receptor antagonist) or imipramine. Stronger therapeutic effect of GD than that of imipramine, fluoxetine, and D-AP7	Interactions with NMDA, serotonin, and/or noradrenergic systems	[178]
**Corm or bulbs**	aqueous and lyophilized extract, macerate	150 mg kg^−1^, p.o./*	Protection against pentylenetetrazol (PTZ)- and maximal electroshock (MES)-induced seizures. Additive effect of co-administration of GD with diazepam, opposite to the combination of GD with flumazenil or FG7142. Sedative activity of GD by shortening the latency time to sleep and an increase in the total duration of diazepam-induced sleep used for evaluation of the sedative properties	Via the benzodiazepine site receptor	[177]
	aqueous extract	7.5 or 15 mg kg^−1^, every day during 28 days, 5 min before induction of stress, p.o./**	Antagonization of the chronic immobilization of stress-induced behavioral, reproductive, and neurochemical changes in female albino rats by the GD extract. Increase in the number of entries and prolonged time of open arm exploration in the elevated plus maze (EPM). Reduction of the corticosterone, progesterone, and prolactin concentrations elevated due to chronic stress as well as normalization of the level of reproductive hormones and reversed unfavorable changes in the estrous cycle by GD	Possible role of the bioactive molecules and secondary metabolites (alkaloids, flavonoids) in the potential adaptogenic action of GD against a chronic restraint model in animals. Plausible mediation of GD action through interactions with NMDA, GABA, 5-HT and/ or NA systems	[273]

Explanations: ACTH—adrenocorticotropin, corticotropin; GD—*Gladiolus dalenii*. See also the explanations to Table 1.
